# Smoking, alcohol consumption, diabetes, body mass index, and peptic ulcer risk: A two-sample Mendelian randomization study

**DOI:** 10.3389/fgene.2022.992080

**Published:** 2023-01-06

**Authors:** Yi Liu, Zhihan Xiao, Kun Ye, Linlin Xu, Yanping Zhang

**Affiliations:** ^1^ Department of Digestive System, Anqing Municipal Hospital, Anqing, China; ^2^ Department of Digestive System, Wannan Medical College, Wuhu, China; ^3^ Department of Thoracic Surgery, The First Affiliated Hospital of Nanjing Medical University, Nanjing, China

**Keywords:** smoking, alcohol consumption, diabetes, body mass index, peptic ulcer, Mendelian randomization

## Abstract

**Background:** Observational evidence has shown that smoking, alcohol consumption, type 2 diabetes, and body mass index (BMI) are risk factors for peptic ulcer disease (PUD), including gastric ulcer (GU) and duodenal ulcer (DU). However, the observed associations may be confounding factors. Herein, we use Mendelian randomization (MR) to examine causal associations such as smoking, alcohol, type 2 diabetes, BMI, and risks of PUD.

**Methods:** We used 8,17,41,325,82, 231, and 616 identified genetic variants as proxies for age of smoking initiation (AgeSmk), smoking cessation (SmkCes, current/former), number of cigarettes smoked per day (CigDay), smoking status (SmkIni, ever/never), alcohol consumption, type 2 diabetes, and BMI to obtain unconfounded effect estimates on the GU and DU levels among 452,264 participants from the Gene ATLAS. The causal relationship was estimated by using inverse-variance weighted (IVW) as the main method. Sensitivity analysis includes Cochran’s Q test, the MR-Egger test, MR pleiotropy residual sum and outlier (MR-PRESSO), and MR-robust adjusted profile score (MR-RAPS). In addition, secondary MR analysis was conducted within summary data using genetic risk scores (GRSs) as instrumental variables (IVs).

**Results:** In our two-sample MR analyses, genetic predisposition to smoking (SmkInit) and BMI were associated with an increased risk of GU. The beta values were 0.0035 (95% CI, 0.0021, 0.0049, *p* = 1.56E-06) for smoking (SmkInit) and 0.0021 (95% CI, 0.0009, 0.0033, *p* = 0.0008) for BMI. Genetic predisposition to smoking (SmkInit) and higher genetically predicted BMI were associated with an increased risk of DU. The beta values of DU were 0.0029 (95% CI, 0.0017, 0.0041, *p* = 2.43E-06) for smoking (SmkInit) and 0.0018 (95% CI, 0.0007, 0.0029, *p* = 0.001) for BMI. No other causal association between smoking (AgeSmk, CigDay, and SmkCes), alcohol consumption, type 2 diabetes, and GU or DU was observed. Consistent results were obtained in sensitivity analyses. Furthermore, the GRS approach showed similar results in the several MR methods.

**Conclusion:** These findings do not support a causal role of AgeSmk, CigDay, SmkCes, alcohol consumption, and type 2 diabetes in the development of GU and DU. However, it is confirmed that SmkInit and BMI have a causal part in the development of GU and DU.

## 1 Introduction

Symptoms of the common gastrointestinal condition known as peptic ulcer disease (PUD) include nausea, vomiting, abdominal pain, and even bleeding or perforation in the digestive tract. PUD often develops in the proximal duodenum and stomach, but it can also occur in the esophagus, distal duodenum, or jejunum (Lanas and Chan 2017). PUD is generally referred to in clinical medicine primarily as gastric ulcer (GU) or duodenal ulcer (DU), and therefore, GU and DU are collectively referred to as PUD in this study. According to estimates, the overall population’s lifetime prevalence of PUD is between 5 and 10 percent, with an annual incidence of between 0.1 and 0.3 percent (Kurata et al., 1992; Rosenstock and Jørgensen 1995). In the previous 30 years, PUD-related morbidity and mortality have dramatically decreased, whereas in the last 15 years, there has been a progressive rising trend (Xie et al., 2022).

The pathogenesis of PUD is multifactorial and may be related to genetic and environmental factors. In previous studies, it was proposed that smoking (Levenstein et al., 1997; Shigemi et al., 1999; Andersen et al., 2000; Rosenstock et al., 2003), alcohol consumption (Levenstein et al., 1997; Andersen et al., 2000; Rosenstock et al., 2003), type 2 diabetes (Tseng et al., 2012), and BMI (Holtmann et al., 1992; Medalie et al., 1992; Boylan et al., 2014) may be possible elements for the development of PUD. The majority of the data on potential risk factors for PUD are based on observational research, which can be subject to problems from residual confounding. We now evaluate the causal association between smoking, alcohol consumption, type 2 diabetes, BMI, and the likelihood of developing GU and DU using Mendelian randomization (MR).

The drawbacks of observational studies, such as reverse causality, residual confounding, and recall bias, have been overcome by MR analysis, in which genetic variants are used as a surrogate for lifestyle/environmental exposures. Consequently, it is a sophisticated tool for enhancing causal inference (Vandebergh et al., 2022). The random distribution of genetic variants at conception and the fixed status of genetic variants that cannot be changed by the onset or progression of the disease, respectively, allow for the reduction of residual confounding and the diminishing of reverse causality, which are two main benefits of this method (Yuan et al., 2021). Here, we did a two-sample MR study to examine the relationships between the risk of GU and DU and genetically proxied smoking, alcohol consumption, type 2 diabetes, and BMI.

## 2 Materials and methods

### 2.1 Genetic instruments for smoking, alcohol consumption, type 2 diabetes, and BMI

The most recent meta-analysis on tobacco and alcohol consumption based on over 30 GWASs identified 566 and 99 genetic variants associated with four smoking phenotypes and alcohol consumption phenotypes in over 1.2 million people of European ancestry. These genetic instruments for smoking phenotypes and alcohol consumption were obtained from this study (Liu et al., 2019). There have been several descriptions of smoking and alcohol consumption phenotypes in detail (Liu et al., 2019). In summary, the smoking status (SmkIni, Ever/Never), age at smoking initiation (AgeSmk), smoking cessation (SmkCes, Current/Former), and number of cigarettes smoked per day (CigDay, both for current and former smokers, quantitative measures were binned into five bins or collected with predefined bins as follows: 1 = 1–5, 2 = 6–15, 3 = 16–25, 4 = 26–35, and 5 = 36 + cigarettes per day) were included (Zhou et al., 2021). For all phenotypes in family studies and quasi-continuous phenotypes (age of smoking initiation and cigarettes per day) in unrelated individuals, the association data were produced using a linear mixed model (Zhou et al., 2021). In research of unrelated individuals, a logistic model was used to calculate the additive genetic effects for the binary phenotypes of the smoking status and smoking cessation. Meta-analysis was carried out using rareGWAMA (McKay et al., 2017). Supplementary Tables S1–S5 provide comprehensive data regarding instrumental variables (IVs) related to alcohol intake and smoking phenotypes.

Based on a meta-analysis of 32 GWASs including 74,124 type 2 diabetes cases and 824,006 control people of European ancestry, the IVs selection for type 2 diabetes was conducted (known as the DIAGRAM consortium) (Mahajan et al., 2018). Single-nucleotide polymorphisms (SNPs) were identified as IVs for type 2 diabetes (*n* = 403) when they reached the genome-wide statistical significance criteria (*p* < 5 * 10^–8^). According to earlier research, BMI-mediation effects were entirely for the impacts of type 2 diabetes-related genetic variations in the FTO, MC4R, TMEM18, SEC16B, and GNPDA2 genes (Mahajan et al., 2018). The FTO, MC4R, TMEM18, SEC16B, and GNPDA2 gene regions were thus removed, leaving 394 SNPs as type 2 diabetes instrumental factors. In the sensitivity analysis for type 2 diabetes, 289 SNPs that reached the genome-wide significance threshold were included (FTO, MC4R, TMEM18, SEC16B, and GNPDA2 variations were removed). In Supplementary Table S6, comprehensive details for type 2 diabetes IVs are provided.

The exposure variable data for the genetic variants associated with BMI was derived from a GWAS meta-analysis in the Genetic Investigation of Anthropometric Traits (https://portals.broadinstitute.org/collaboration/giant/index.php/GIANT_consortium_data_files, accessed on 28 April 2021) consortium (*n* = 681,275 individuals of European ancestry) (Yengo et al., 2018). Detailed information for IVs of BMI is presented in Supplementary Table S7.

### 2.2 Genetic associations of SNPs with gastric ulcer and duodenal ulcer risk

Summary statistics on GU and DU risks, including standard errors (SE) and odds ratio (OR) estimates for instrumental SNPs, were available from Gene ATLAS (http://geneatlas.roslin.ed.ac.uk/) in 452,264 participants from European ancestry. Participants with more derived European ancestry, as indicated by these studies, were only allowed to participate.

### 2.3 Instrumental variable selection

Based on the following criteria, the IVs for MR studies were chosen: 1) a 500-kb window with *r* ^2^ measure of LD among instruments <0.001; 2) *p* value lower than the genome-wide significant level (5 * 10^–8^ for smoking, alcohol consumption, type 2 diabetes, and BMI) found in the relevant study; 3) minor allele frequency (MAF) > 0.01; 4) palindromic SNPs (A/T and G/C polymorphisms were considered for elimination (MAF is between 0.4 and 0.6), but there was no ambiguity in the effects).

### 2.4 Statistical analyses

#### 2.4.1 Mendelian randomization analysis

**FIGURE 1:**
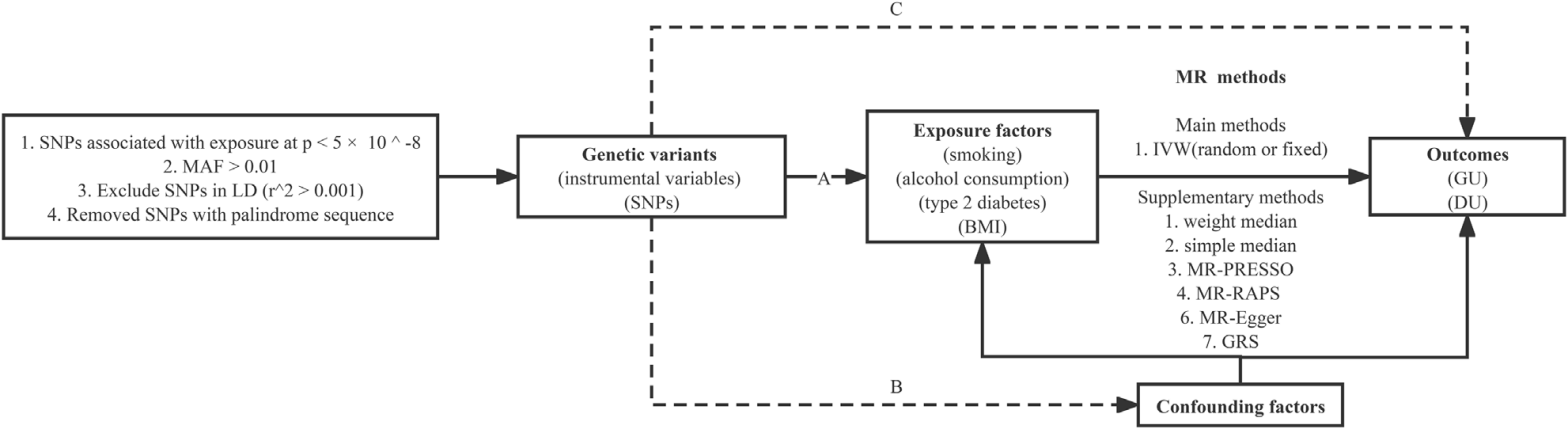
Study design overview and assumptions of the Mendelian randomization framework. IVW, inverse-variance weighted; LD, linkage disequilibrium; SNPs, single-nucleotide polymorphisms. MR-PRESSO, Mendelian randomization pleiotropy residual sum and outlier; MR-RAPS, Mendelian randomization robust-adjusted profile score. It is shown that **(A)** the genetic variants proposed as instrumental variables should be robustly associated with the risk factor of interest; **(B)** the used genetic variants should not be associated with potential confounders; and **(C)** the selected genetic variants should affect the risk of the outcome merely through the risk factor, not *via* alternative pathways.

In our two-sample MR study, we used the inverse-variance weighted (IVW) approach as the main method to assess the causal relationship of smoking, alcohol consumption, type 2 diabetes, and BMI with GU and DU. IVW calculates the exposure-outcome effect corresponding to each SNP using the Wald ratio method, then performs a weighted linear regression with a forced intercept of zero. It achieved higher estimate accuracy and test power when IVs satisfied the following three underlying assumptions (Burgess et al., 2013): 1) the SNPs are significantly associated with the exposure (the “relevance” assumption); 2) the SNPs are not correlated with potential confounders of the exposure-outcome relation (the “exchangeability” assumption); and 3) the SNPs are not directly associated with the outcome (the “exclusion” restriction) (Figure 1). If there was significant heterogeneity, a random-effects IVW model would be implemented, which was less prone to the bias of weaker SNP-exposure associations (Bowden et al., 2017). To avoid the interference of unknown and unmeasurable confounders, we also performed the MR-Egger regression (MR-Egger) (Bowden et al., 2015), weighted median estimator (Verbanck et al., 2018b), simple median estimator (Verbanck et al., 2018b), MR pleiotropy residual sum and outlier (MR-PRESSO) test (Verbanck et al., 2018a), and the robust adjusted profile score (RAPS) (Zhao et al., 2020a) to test the robustness of our results. The study design overview is shown in Figure 1.

The Bonferroni method was used to correct for multiple testing in our two-sample MR models. The association with two-sided *p* values <0.007 (where *α* = 0.05/7) were deemed statistically significant. Associations with *p* values between 0.05 and 0.007 were regarded as suggestive associations, requiring confirmation. We conducted all analyses using R (version 4.1.2) the R packages “TwoSampleMR” (Hemani et al., 2018) and “MendelianRandomization” (Yavorska and Burgess 2017). We used only freely accessible summarized data in this study; therefore, this work did not require ethical approval.

#### 2.4.2 Associations between exposure genetic risk score (GRS) and outcome

MR analysis was carried out using weighted GRS as IVs by using the same summary data to produce the combined estimate of the connection between exposure-influencing alleles and the outcome. We used R (version 4.1.2) and the “gtx” R package (Windows version 0.0.8), whose grs. summary module contains the GRS function, to carry out the analyses. Similar to an approach where an outcome is regressed onto an additive GRS, the grs. summary module only used single SNP association summarized data from the outcomes of the GWAS research (Luo et al., 2020). This method is explained in depth elsewhere (Dastani et al., 2012). Additionally, prior research claimed that this MR approach using meta-GWAS summary data was just as effective as that using individual-level data (Voight et al., 2012).

#### 2.4.3 Sensitivity analyses

##### 2.4.3.1 Weighted median estimates

We provide weighted median estimates, which are accurate estimates when at least 50% of the data comes from reliable SNPs (Verbanck et al., 2018b).

##### 2.4.3.2 MR-Egger

If the instrument strength independent of direct effect (InSIDE) condition is met, MR-Egger regression yields accurate estimates even when all the SNPs are incorrect (Bowden et al., 2015). The MR-Egger method relaxes the requirement that there is no pleiotropy between genetic variants in the IVW method. It assumes that the IVs–exposure and IVs–outcome associations are independent. This is referred to as the InSIDE assumption and is relatively weak compared to the strict exclusion restriction criteria. MR-Egger intercepts with *p* < 0.05, indicating that horizontal pleiotropy are present. Horizontal pleiotropy refers to the effect of genetic variation on a trait through multiple different paths. When using pleiotropy genetic variation as IVs, it is equivalent to constructing other pathways other than the “IVs–exposure–outcome” path, thus making the IVs fail due to the breach of core assumptions. The application of pleomorphic IVs can lead to bias in the estimation of the effect of the studied causal pathway. We concentrated more on the consistency of the estimate direction between MR-Egger and IVW because MR-Egger has less statistical power than IVW (Yeung et al., 2019).

##### 2.4.3.3 MR pleiotropy residual sum and outlier (MR-PRESSO)

By examining outliers among the included SNPs that contribute to the MR estimate, MR-PRESSO can identify horizontal pleiotropy. This approach makes the assumptions that the InSIDE assumption is true, balanced pleiotropy, and that at least 50% of the SNPs are valid SNPs. Additionally, MR-PRESSO offers adjusted estimates and identifies the outlier SNPs. This approach was used by us as a statistical tool to detect and eliminate potential pleiotropic SNPs (Bowden et al., 2016). In summary, the MR-PRESSO method has several core functions: 1) to detect the presence of horizontal pleiotropy using the “MR-PRESSO global test,” MR-PRESSO global test *p* < 0.05 indicates the presence of horizontal pleiotropy; 2) to remove abnormal SNPs (outliers) and estimate the corrected results (which remove horizontal pleiotropy) using the “MR-PRESSO outlier test.” The MR-PRESSO (raw) method is the result before the removal of the abnormal SNPs, and MR-PRESSO (outlier-corrected) is the result after the removal of the abnormal SNPs. When the results of the MR-PRESSO (outlier-corrected) method show “NA,” this indicates that there are no outlier SNPs in the IVs we selected.

##### 2.4.3.4 MR-robust adjusted profile score (MR-RAPS)

The MR-RAPS method corrected for horizontal pleiotropy in the IVW analysis by using robust-adjusted profile scores. In addition, MR-RAPS with Huber loss function which can model random-effects distribution of the pleiotropic effects of genetic variants is discussed (Zhao et al., 2020b).

##### 2.4.3.5 Cochran’s Q test

To assess the extent of heterogeneity among the individual impact estimates produced from each genetic mutation, the Cochran’s Q test was calculated (Haycock et al., 2016). Cochran’s Q statistic was used to determine heterogeneity statistics. Heterogeneity was defined as a *p* value <0.05 from a Cochran’s Q calculation. A random-effects IVW model would be used if there was high heterogeneity since it was less susceptible to the bias of weaker SNPs-exposure relationships (Bowden et al., 2017).

##### 2.4.3.6 F statistics

In order to detect the strength of the IVs at a threshold of F > 10, which is normally advocated in MR analysis, we calculated the F statistics of the identified all SNPs. The formula for the F statistics is $F=\frac{R^{2}\left(n-1-K\right)}{\left(1-R^{2}\right)K}$ , in this equation, K corresponds to the number of SNPs, n to the number of exposures, and *R* ^2^ to the amount of variation explained by the SNPs (Pierce and Burgess 2013).

## 3 Results

### 3.1 Instrumental variables and their validity

A total of 231 independent SNPs for type 2 diabetes, 82 independent SNPs for alcohol consumption, 616 independent SNPs for BMI, and 8, 41, 17, and 325 SNPs for the AgeSmk, CigDay, SmkCes, and SmkInit of smoking phenotypes, respectively, were included as IVs (Supplementary Tables S1–S7).

### 3.2 Mendelian randomization

#### 3.2.1 MR results of smoking to GU and DU

Genetic predisposition to SmkInit was associated with an increased risk of GU and DU. These associations are consistent across multiple MR methods. The beta and corresponding 95% CIs of GU were 0.0035 (95% CI, 0.0021, 0.0049) for one SD increase for SmkInit and DU were 0.0029 (95% CI, 0.0017, 0.0041) for one SD increase for SmkInit (Table 1, Supplementary Figures S1–S2). Genetic predisposition to AgeSmk, CigDay, and SmkCes was not associated with GU and DU susceptibilities (Table 1, Supplementary Figures S1–S2).

**TABLE 1:** Summarized results of the Mendelian randomization study on smoking, alcohol consumption, diabetes, and BMI and GU and DU risks.

Group	MR approach	GU				DU			
		Beta	95% CI		*p* value	Beta	95% CI		*p* value
AgeSmk	IVW(random)	0.0029	-0.0066	0.0123	0.55	-0.0037	-0.0132	0.0059	0.45
	IVW(fix)	0.0029	-0.0066	0.0123	0.55	-0.0037	-0.0117	0.0044	0.37
	Simple median	0.0018	-0.0100	0.0135	0.77	-0.0081	-0.0193	0.0031	0.16
	Weighted median	0.0030	-0.0090	0.0149	0.63	0.0004	-0.0111	0.0119	0.95
	MR-RAPS	0.0029	-0.0069	0.0127	0.56	-0.0038	-0.0120	0.0044	0.37
	MR-PRESSO (raw)	0.0029	-0.0033	0.0091	0.39	-0.0037	-0.0132	0.0059	0.48
	MR-PRESSO (outlier-corrected)	NA	NA	NA	NA	NA	NA	NA	NA
	MR-Egger	0.0247	-0.0243	0.0737	0.32	0.0493	0.0075	0.0912	0.02
CigDay	IVW(random)	0.0012	-0.0016	0.0041	0.40	0.0020	-0.0007	0.0047	0.14
	IVW(fix)	0.0012	-0.0016	0.0041	0.40	0.0020	-0.0004	0.0045	0.10
	Simple median	0.0024	-0.0028	0.0076	0.36	0.0014	-0.0030	0.0057	0.53
	Weighted median	-0.0011	-0.0054	0.0032	0.62	0.0005	-0.0032	0.0042	0.79
	MR-RAPS	0.0013	-0.0016	0.0042	0.40	0.0021	-0.0004	0.0046	0.10
	MR-PRESSO (raw)	0.0012	-0.0015	0.0040	0.38	0.0020	-0.0007	0.0047	0.15
	MR-PRESSO (outlier-corrected)	NA	NA	NA	NA	NA	NA	NA	NA
	MR-Egger	-0.0014	-0.0065	0.0037	0.59	0.0018	-0.0031	0.0066	0.47
SmkCes	IVW(random)	0.0005	-0.0028	0.0038	0.76	0.0009	-0.0020	0.0037	0.55
	IVW(fix)	0.0005	-0.0028	0.0038	0.76	0.0009	-0.0020	0.0037	0.55
	Simple median	0.0006	-0.0045	0.0057	0.82	0.0022	-0.0021	0.0066	0.31
	Weighted median	-0.0003	-0.0049	0.0043	0.89	0.0020	-0.0020	0.0060	0.32
	MR-RAPS	0.0005	-0.0028	0.0038	0.76	0.0009	-0.0020	0.0038	0.55
	MR-PRESSO (raw)	0.0005	-0.0022	0.0032	0.72	0.0009	-0.0019	0.0036	0.55
	MR-PRESSO (outlier-corrected)	NA	NA	NA	NA	NA	NA	NA	NA
	MR-Egger	-0.0065	-0.0156	0.0026	0.16	-0.0039	-0.0117	0.0039	0.33
SmkInit	IVW(random)	0.0035	0.0021	0.0049	1.56E-06	0.0029	0.0017	0.0041	2.43E-06
	IVW(fix)	0.0035	0.0021	0.0049	3.67E-07	0.0029	0.0018	0.0041	7.57E-07
	Simple median	0.0050	0.0029	0.0070	2.12E-06	0.0029	0.0011	0.0046	1.26E-03
	Weighted median	0.0041	0.0020	0.0062	1.02E-04	0.0030	0.0013	0.0048	7.11E-04
	MR-RAPS	0.0036	0.0022	0.0050	3.37E-07	0.0030	0.0018	0.0042	7.09E-07
	MR-PRESSO (raw)	0.0035	0.0021	0.0049	2.38E-06	0.0029	0.0017	0.0041	3.61E-06
	MR-PRESSO (outlier-corrected)	NA	NA	NA	NA	NA	NA	NA	NA
	MR-Egger	0.0011	-0.0049	0.0071	0.72	0.0018	-0.0033	0.0069	0.48
DrnkWk	IVW(random)	-0.0030	-0.0080	0.0021	0.25	0.0027	-0.0016	0.0070	0.21
	IVW(fix)	-0.0030	-0.0078	0.0018	0.23	0.0027	-0.0014	0.0069	0.20
	Simple median	0.0001	-0.0071	0.0072	0.98	0.0012	-0.0050	0.0075	0.70
	Weighted median	-0.0009	-0.0080	0.0063	0.81	0.0001	-0.0061	0.0063	0.97
	MR-RAPS	-0.0030	-0.0079	0.0019	0.22	0.0028	-0.0015	0.0071	0.20
	MR-PRESSO (raw)	-0.0030	-0.0080	0.0021	0.25	0.0027	-0.0016	0.0070	0.22
	MR-PRESSO (outlier-corrected)	NA	NA	NA	NA	NA	NA	NA	NA
	MR-Egger	-0.0109	-0.0283	0.0066	0.22	-0.0103	-0.0248	0.0043	0.17
Diabetes	IVW(random)	0.0002	-0.0003	0.0007	0.46	0.0002	-0.0002	0.0007	0.29
	IVW(fix)	0.0002	-0.0003	0.0007	0.43	0.0002	-0.0002	0.0006	0.24
	Simple median	0.0006	-0.0002	0.0014	0.14	0.0007	0.0000	0.0014	0.06
	Weighted median	0.0003	-0.0006	0.0012	0.47	-0.0001	-0.0008	0.0006	0.80
	MR-RAPS	0.0002	-0.0002	0.0006	0.43	0.0002	-0.0002	0.0007	0.24
	MR-PRESSO (raw)	0.0002	-0.0003	0.0007	0.46	0.0002	-0.0002	0.0007	0.29
	MR-PRESSO (outlier-corrected)	NA	NA	NA	NA	NA	NA	NA	NA
	MR-Egger	0.0002	-0.0008	0.0012	0.74	-0.0003	-0.0011	0.0006	0.56
BMI	IVW(random)	0.0021	0.0009	0.0033	0.001	0.0018	0.0007	0.0029	0.001
	IVW(fix)	0.0021	0.0009	0.0033	0.001	0.0018	0.0008	0.0028	0.0006
	Simple median	0.0022	0.0004	0.0040	0.02	0.0015	-0.0001	0.0031	0.06
	Weighted median	0.0018	-0.0003	0.0040	0.10	0.0027	0.0009	0.0045	0.003
	MR-RAPS	0.0021	0.0009	0.0033	0.0006	0.0018	0.0008	0.0028	0.0006
	MR-PRESSO (raw)	0.0021	0.0009	0.0033	0.001	0.0018	0.0007	0.0029	0.001
	MR-PRESSO (outlier-corrected)	NA	NA	NA	NA	NA	NA	NA	NA
	MR-Egger	0.0009	-0.0025	0.0043	0.61	0.0046	0.0016	0.0077	0.003

Summarized results of the Mendelian randomization study on smoking, alcohol consumption, type 2 diabetes, and BMI on GU and DU risks. AgeSmk, age of smoking initiation; CigDay, number of cigarettes smoked per day; SmkCes, smoking cessation (Current/Former); SmkInit, smoking status (Ever/Never); DrnkWk, drinks per Week; BMI, body mass index; GU, gastric ulcer; DU, duodenal ulcer; IVW, inverse-variance weighted; MR-PRESSO, Mendelian randomization pleiotropy residual sum and outlier; MR-RAPS, Mendelian randomization robust adjusted profile score.

#### 3.2.2 MR results of alcohol consumption to GU and DU

Increased alcohol consumption had no discernible influence on the risk of GU and DU (beta = -0.0030, 95% CI = -0.0080, 0.0021, *p* = 0.25; beta = 0.0027, 95% CI = -0.0016, 0.0070, *p* = 0.21, respectively) (Table 1). The MR-Egger regression and Cochran’s Q test did not reveal any evidence that supported horizontal pleiotropy and heterogeneity. MR-PRESSO shows an SNP with horizontal pleiotropic, with one SNP identified as outliers (rs281379). When we removed rs281379, our results were stable, and neither horizontal pleiotropic nor heterogeneity was detected (Table 3). The results of all methods were consistent, and MR-PRESSO did not show the existence of pleiotropic SNPs (Table 1, Supplementary Figures S1–S2).

#### 3.2.3 MR results of type 2 diabetes to GU and DU

Genetic predisposition to type 2 diabetes was not associated with an increased risk of GU and DU. These associations are consistent across multiple MR methods. The beta and corresponding 95% CIs of GU were 0.0002 (95% CI, -0.0003, 0.0007) for type 2 diabetes and DU were 0.0002 (95% CI, -0.0002, 0.0007) for type 2 diabetes (Table 1, Supplementary Figures S1–S2). The MR-Egger regression intercept provided no support for directional pleiotropy (Table 3). The Cochran’s Q test did show some heterogeneity among individual SNPs effect estimates in type 2 diabetes on DU; therefore, a random-effects IVW model would be implemented. Random-effects IVW model results were consistent with other methods (fixed-effects IVW model weighted median estimator, simple median estimator, MR-RAPS, and MR-PRESSO) (Table 1, Supplementary Figures S1–S2).

#### 3.2.4 MR results of BMI to GU and DU

There was a link between a higher risk of GU and DU and a genetic tendency to BMI. The beta and corresponding 95% CIs of GU were 0.0021 (95% CI, 0.0009, 0.0033) for one SD increase for BMI and DU were 0.0018 (95% CI, 0.0007, 0.0029) for one SD increase for BMI (Table 1, Supplementary Figures S1–S2). The Cochran’s Q test and MR-Egger regression intercept for the relationship between BMI and GU did not reveal any evidence of directional pleiotropy or heterogeneity, and the Cochran’s Q test and MR-Egger regression intercept did show heterogeneity and directional pleiotropy among individual SNPs effect estimates in BMI on DU (Table 3). However, no SNPs were identified as an outlier in the MR-PRESSO method in the BMI to DU MR analysis. In addition, random-effects IVW model results were consistent with other methods (fixed-effects IVW model, weighted median estimator, simple median estimator, MR-RAPS, and MR-PRESSO) (Table 1, Supplementary Figures S1–S2).

### 3.3 GRS_smoking,_ GRS_alcohol,_ GRS_type 2 diabetes_, and GRS_BMI_ with GU and DU

Consistent with the MR results of smoking (AgeSmk, CigDay, SmkCes, and Smklnit), alcohol consumption, type 2 diabetes, and BMI to GU and DU, the GRS revealed no causal effect of smoking (AgeSmk, CigDay, and SmkCes), alcohol consumption, and type 2 diabetes on GU and DU risks (Table 2). The GRS revealed significant causal effect of smoking (Smklnit) and BMI on GU and DU (Table 2). The beta and corresponding 95% CIs of GU were 0.0035 (95% CI, 0.0022, 0.0048) for one SD increase for Smklnit and DU were 0.0029 (95% CI, 0.0018, 0.0041) for one SD increase for Smklnit (Table 2, Supplementary Figure S3). The beta and corresponding 95% CIs of GU were 0.0021 (95% CI, 0.0009, 0.0033) for one SD increase for BMI and DU were 0.0018 (95% CI, 0.0008, 0.0028) for one SD increase for BMI (Table 2, Supplementary Figure S3).

**TABLE 2:** Effect of the GRS instrument of smoking, alcohol consumption, diabetes, and BMI on GU, DU.

Exposure	Outcome	Beta	95% CI		*p* value
AgeSmk	GU	0.0029	-0.0066	0.0123	0.55
	DU	-0.0037	-0.0117	0.0044	0.37
CigDay	GU	0.0012	-0.0016	0.0041	0.40
	DU	0.0020	-0.0004	0.0045	0.10
SmkCes	GU	0.0005	-0.0028	0.0038	0.76
	DU	0.0009	-0.0020	0.0037	0.55
SmkInit	GU	0.0035	0.0022	0.0048	3.67E-07
	DU	0.0029	0.0018	0.0041	7.57E-07
DrnkWk	GU	-0.0030	-0.0078	0.0018	0.23
	DU	0.0027	-0.0014	0.0069	0.20
Diabetes	GU	0.0002	-0.0003	0.0007	0.43
	DU	0.0002	-0.0002	0.0006	0.24
BMI	GU	0.0021	0.0009	0.0033	6.31E-04
	DU	0.0018	0.0008	0.0028	5.97E-04

The effect of the GRS, instrument of smoking, alcohol consumption, type 2 diabetes, and BMI on GU and DU AgeSmk, age of smoking initiation; CigDay, number of cigarettes smoked per day; SmkCes, smoking cessation (Current/Former); SmkInit, smoking status (Ever/Never); DrnkWk, drinks per Week; BMI, body mass index; GU, gastric ulcer; DU, duodenal ulcer.

### 3.4 Heterogeneity and sensitivity analysis

Cochran’s Q tests revealed that there was no heterogeneity between the smoking, alcohol consumption, type 2 diabetes, and BMI IVs in the study of smoking, alcohol consumption, type 2 diabetes, BMI, and GU MR (Table 3). In smoking and alcohol consumption to DU MR analysis, the results of Cochran’s Q tests showed that there was no heterogeneity among the smoking and alcohol consumption-related IVs (Table 3). In type 2 diabetes and BMI to DU MR analysis, the Cochran’s Q tests revealed some heterogeneity between both the IVs for type 2 diabetes (Q = 284.7, *p* = 0.01) and BMI (Q = 689.6, *p* = 0.02) (Table 3); however, random-effects IVW model results were consistent with other methods (fixed-effects IVW model, weighted median estimator, simple median estimator, MR-RAPS, and MR-PRESSO). Therefore, our results are still very reliable and this little heterogeneity has little effect on our results.

**TABLE 3:** Potential pleiotropy evaluation using different methods.

Exposure	Outcome	SNPs	Cochran’s Q statistic	Cochran’s Q p	MR-Egger p	MR-PRESSO global test	MR-PRESSO global test p
AgeSmk	GU	8	2.994	0.89	0.37	3.914	0.89
	DU	8	9.739	0.20	0.01	12.786	0.22
CigDay	GU	41	36.202	0.64	0.22	39.754	0.60
	DU	41	49.022	0.16	0.90	51.198	0.18
SmkCes	GU	17	10.834	0.82	0.11	12.664	0.81
	DU	17	15.075	0.52	0.20	17.515	0.50
SmkInit	GU	325	363.039	0.07	0.42	365.283	0.07
	DU	325	356.656	0.10	0.67	358.940	0.11
DrnkWk	GU	82	88.964	0.26	0.35	91.448	0.24
	DU	81	85.352	0.32	0.07	87.509	0.32
Diabetes	GU	231	263.379	0.06	0.96	266.542	0.06
	DU	231	284.719	0.01	0.19	287.024	0.01
BMI	GU	616	633.806	0.29	0.46	635.844	0.29
	DU	616	689.561	0.02	0.049	691.821	0.02

Potential pleiotropy evaluation using different methods. AgeSmk, age of smoking initiation; CigDay, number of cigarettes smoked per day; SmkCes, smoking cessation (Current/Former); SmkInit, smoking status (Ever/Never); DrnkWk, drinks per Week; BMI, body mass index; GU, gastric ulcer; DU, duodenal ulcer; SNPs**,** single-nucleotide polymorphisms; and MR-PRESSO, Mendelian randomization pleiotropy residual sum and outlier.

The MR-Egger regression analysis revealed that alcohol consumption, type 2 diabetes, smoking, and BMI to GU MR did not indicate horizontal pleiotropy of the IVs (Table 3), and the horizontal pleiotropy of the IVs was not present for alcohol consumption, smoking (CigDay, SmkCes, and Smklnit), and type 2 diabetes to DU MR and it was present for smoking (AgeSmk, MR-Egger *p* = 0.01) and BMI (MR-Egger *p* = 0.049) (Table 3). The MR-PRESSO global test *p* was >0.05 in smoking (AgeSmk) and global test *p* was <0.05 in BMI to DU MR, with no SNPs identified as outliers (Table 3).

### 3.5 Excluding associations between individual exposure factors

To exclude associations between individual exposure factors, we searched the phenoscanner website for SNPs of each exposure factor (Kamat et al., 2019). SNPs that were strongly correlated with other exposure factors were eliminated. Specific screening results of SNPs for each exposure factor are presented in Supplementary Table S8. After removing these SNPs, which were strongly correlated with other exposure factors, we performed MR analysis again, and we got the same results as before (Supplementary Table S9). Therefore, our results can be considered very reliable.

## 4 Discussion

Increasing evidence indicates the association between smoking, alcohol consumption, type 2 diabetes, and BMI with GU and DU risks, and several plausible mechanisms underlying are proposed. According to several studies, smoking constricts the mucosa’s blood vessels while ischemia lowers the mucosa’s resistance (Andersen et al., 2000). Given that smokers’ levels of carboxyhemoglobin are higher, carbon monoxide may have a further role in the mucosal ischemia (Nolan et al., 1985; Smith et al., 1998). The substantial link between smoking and PUD may be explained by these latter mechanisms. When exposed to high levels of ethanol quickly, the result has significant damage to the gastric epithelium, necrosis of deeper layers of the mucosa, and microvascular damage that causes engorgement, increased permeability, and intramucosal bleeding (Tarnawski et al., 1987). Apart from its irritant properties locally, ethanol has been demonstrated to slow down stomach emptying at moderate to high dosages (Jian et al., 1986). Type 2 diabetes-related angiopathy could compromise mucosal integrity and result in more serious ulcers (Weil et al., 2000). Past researches looked into the relationship between PUD and BMI, but the results were still debatable. Past researches have reported obesity as a separate risk factor for PUD (Aro et al., 2006; Garrow and Delegge 2010; Boylan et al., 2014). Due to the observational study’s design, these researches are still unable to control the impact of potential biases. This has led to growing interest in proving a causal relationship between smoking, alcohol consumption, type 2 diabetes, and BMI with GU and DU risks. Our research is the first largest MR analysis the causal association between smoking, alcohol consumption, type 2 diabetes, and BMI with GU and DU to the best of our knowledge.

According to the results of the current MR study, genetic predisposition to BMI and smoking (Smklnit) both enhance the likelihood of developing GU and DU. However, there is no evidence linking a genetic propensity to smoking (AgeSmk, CigDay, and SmkCes), alcohol consumption, or type 2 diabetes to an increased risk of GU and DU.

The following are this study’s strengths and limitations. First, the MR method, which can reduce reverse causality and diminish residual confounding, is the study’s main strength. Second, to assure an accurate outcome, we chose the largest GWAS databases on smoking, alcohol consumption, type 2 diabetes, and BMI. Third, for the first time, we used MR to draw the conclusion that genetically predicted BMI and smoking (Smklnit) increase the risk of GU and DU, which also offers some reasons for certain earlier epidemiological studies. At last, secondary MR analyses were conducted within summary data using GRSs as IVs, and we got the same result as the previous analysis. However, we also recognize that there are some drawbacks. First, since only people of European ancestry were included in the GWASs from which we chose our IVs, it is difficult to extrapolate our results to other ethnicities. Second, potential pleiotropy is a drawback of our work. Third, we did not conduct further multivariable MR analysis to make our research results more convincing. However, in order to exclude associations between exposure individual factors, we searched the phenoscanner website for the SNPs of each exposure factor, excluded SNPs that were strongly correlated with other exposure factors and product the MR analysis again, which was found to be consistent with the previous results, thus, to some extent, compensating for the lack of multivariable MR analysis.

Summary of findings: In a large, well-powered study, we did not find strong evidence for a possible association for genetically predicted smoking (AgeSmk, CigDay, and SmkCes), alcohol consumption, or type 2 diabetes in the risk of GU and DU. This suggests that previous study may be confounded by potential biases or due to reverse causation. However, we found a causal relationship between smoking (Smklnit) and BMI on the one hand and GU and DU on the other. Nevertheless, our results require further study of large sample data in the future.

## Data Availability

The original contributions presented in the study are included in the article/Supplementary Material; further inquiries can be directed to the corresponding author.
