# Supplementary figures and images for: Smoking, alcohol consumption, diabetes, body mass index, and peptic ulcer risk: A two-sample Mendelian randomization study
Source: Front Genet. 2023 Jan 6;13:992080. doi: 10.3389/fgene.2022.992080 (PMC9852705; doi:10.3389/fgene.2022.992080)

Figure S1

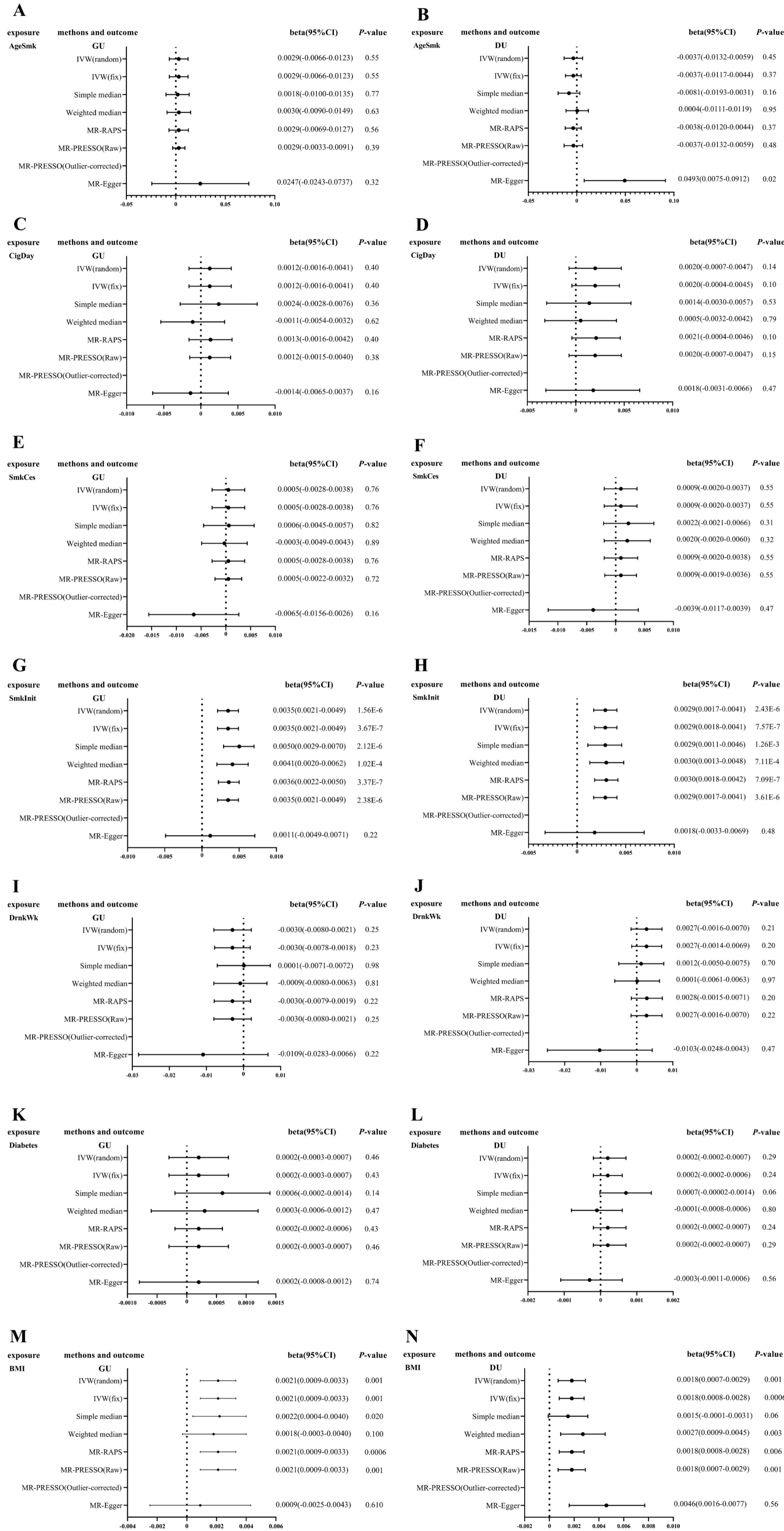

Figure S2

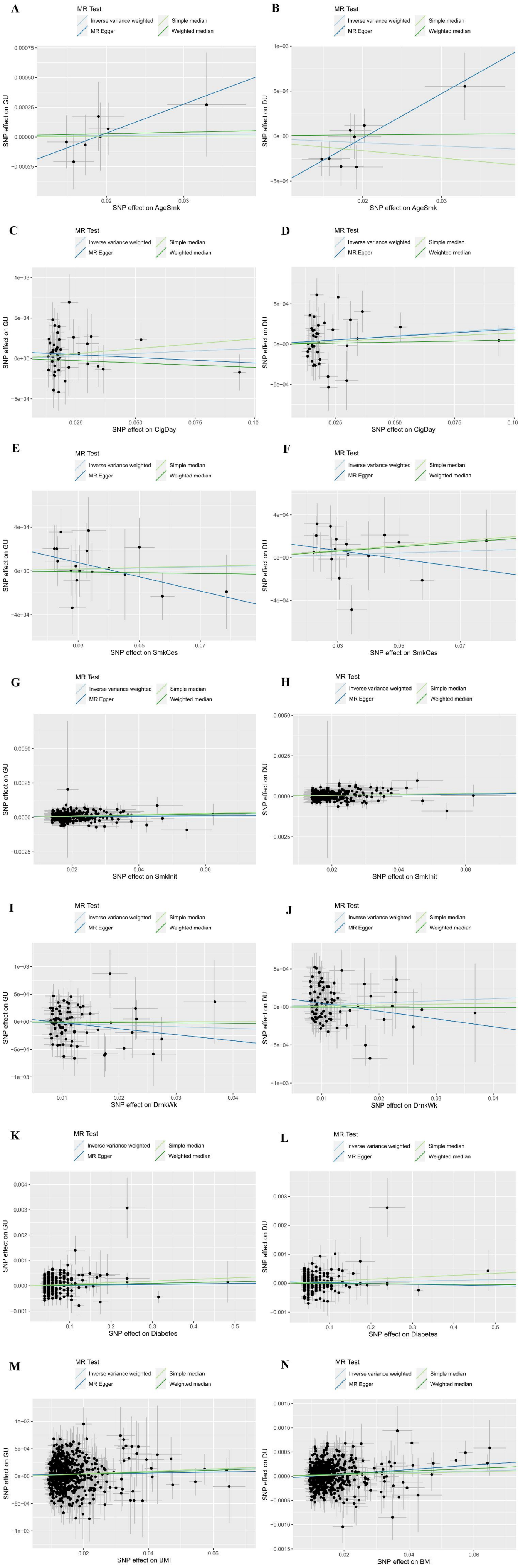

Figure S3

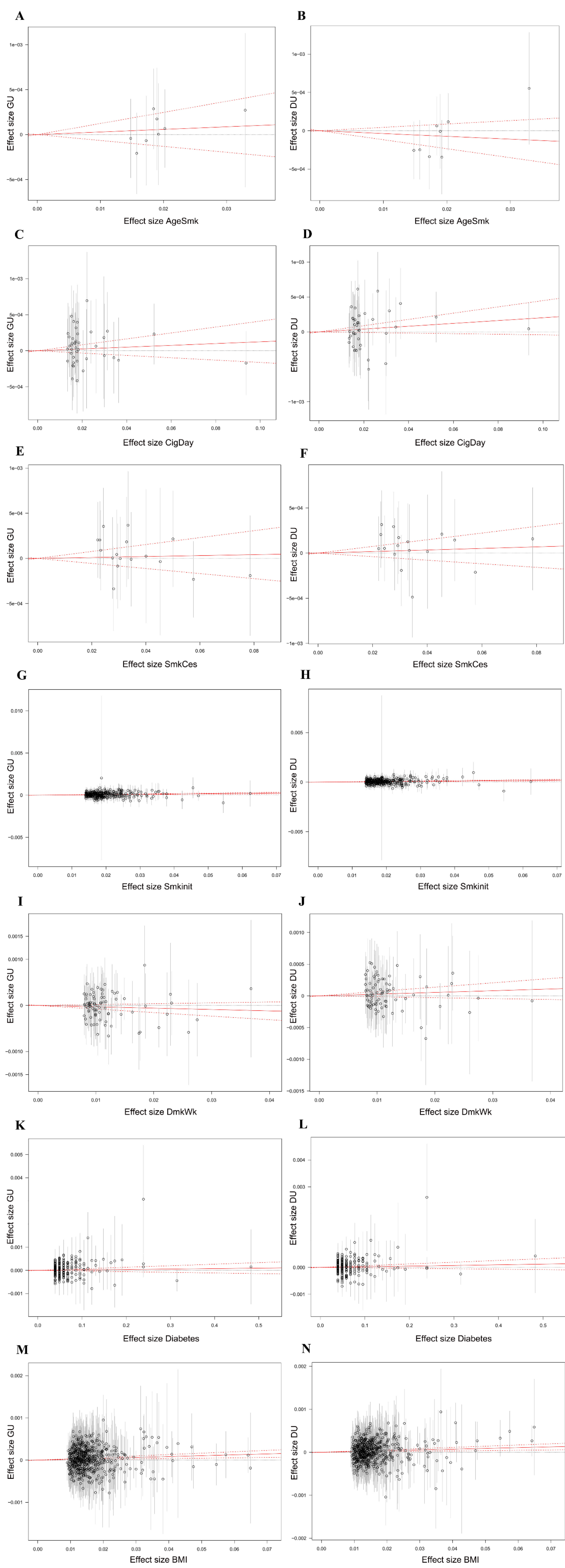

Supplement: Supplementary file 1 [file DataSheet1.PDF]
